# How Mucosal Epithelia Deal with Stress: Role of NKG2D/NKG2D Ligands during Inflammation

**DOI:** 10.3389/fimmu.2017.01583

**Published:** 2017-11-20

**Authors:** Fabrizio Antonangeli, Alessandra Soriani, Cristina Cerboni, Giuseppe Sciumè, Angela Santoni

**Affiliations:** ^1^Department of Molecular Medicine, Sapienza University of Rome, Laboratory Affiliated to Istituto Pasteur Italia – Fondazione Cenci Bolognetti, Rome, Italy; ^2^Neuromed I.R.C.C.S. – Istituto Neurologico Mediterraneo, Pozzilli, Italy

**Keywords:** NKG2D, natural killer cells, toll-like receptor, inflammation, stress, gut, epithelia, innate lymphocytes

## Abstract

Mucosal epithelia encounter both physicochemical and biological stress during their life and have evolved several mechanisms to deal with them, including regulation of immune cell functions. Stressed and damaged cells need to be cleared to control local inflammation and trigger tissue healing. Engagement of the activating NKG2D receptor is one of the most direct mechanisms involved in the recognition of stressed cells by the immune system. Indeed, injured cells promptly express NKG2D ligands that in turn mediate the activation of lymphocytes of both innate and adaptive arms of the immune system. This review focuses on different conditions that are able to modulate NKG2D ligand expression on the epithelia. Special attention is given to the mechanisms of immunosurveillance mediated by natural killer cells, which are finely tuned by NKG2D. Different types of stress, including viral and bacterial infections, chronic inflammation, and cigarette smoke exposure, are discussed as paradigmatic conditions for NKG2D ligand modulation, and the implications for tissue homeostasis are discussed.

## Introduction

Mucosal epithelia represent the frontline of multicellular organisms, and they are continuously exposed to several types of stress. Pathogens and environmental stress (thermal, oxidative, and chemical) can result in cell damage and loss of tissue function. To control inflammation and promote tissue repair, different mechanisms for the detection and elimination of stressed cells have evolved, including activation of the immune system.

NKG2D is a C-type lectin-like activating/co-stimulatory receptor expressed by innate and adaptive lymphocytes, such as natural killer (NK) cells, CD8^+^ αβ T cells, γδ T cells, and iNKT cells. Engagement of NKG2D triggers the cytolytic function of effector CD8^+^ T cells independently of TCR recognition in some circumstances (1–4) while directly activates the effector functions of NK cells, namely, cytolytic granule release and IFNγ secretion. Thus, expression of NKG2D ligands is strictly linked to the immunosurveillance of stressed cells by innate lymphoid cells, especially NK cells (5–7). In humans, NKG2D ligands are MICA and MICB (MHC class I chain-related proteins A and B), encoded by genes in the MHC region, and ULBP1-6 (UL16-binding proteins), with the encoding genes located on chromosome 6. Murine NKG2D ligands include five different isoforms of RAE-1 (retinoic acid early inducible-1), MULT-1 (murine ULBP-like transcript-1), and three different isoforms of H60 (histocompatibility 60) (8). These molecules are present at low or undetectable levels on normal cells (9, 10) but are rapidly induced upon cellular stress and are frequently upregulated in virus-infected and neoplastic cells (11–13). Even if it is known that the promoter region of the *MICA* gene contains a heat-shock element able to respond to cellular stress (14), only recently the molecular mechanisms driving NKG2D ligand expression during cellular stress have more deeply been investigated (15).

Expression of NKG2D ligands has been shown in tissues from patients with chronic inflammatory diseases, including rheumatoid arthritis (16, 17), type 1 diabetes (18), and atherosclerosis (19); much less is known regarding NKG2D ligand expression and relevance in mucosal epithelia during both normal and stress conditions.

This review focuses on different conditions that are able to modulate NKG2D ligands on epithelial cells, and, in particular, on the role of NKG2D/NKG2D ligands in controlling the homeostasis of the gut and lung epithelia during inflammation. Recent findings link toll-like receptor (TLR) signaling to NKG2D ligand expression, and we can now think of epithelial and immune cells as an integrated system able to deal promptly with environmental stress.

## Sensing the Stress: Interplay Between TLRs and NKG2D Ligands

Among the pattern recognition receptors, TLRs play a key role in innate immunity, serving as first line sensors of structurally conserved bacterial and viral components, the so-called pathogen-associated molecular patterns. It is clear that TLRs can be triggered also by endogenous ligands, such as nucleic acids released by necrotic cells and matrix components generated during tissue injury, collectively called damage-associated molecular patterns. As TLR engagement leads to the production of inflammatory cytokines and chemokines, which contribute to local inflammation and leukocyte recruitment, the recent findings showing that TLR signaling is able to modulate the NKG2D axis and thus lymphocyte activation are of great interest (Table 1).

**Table 1:** Modulation of NKG2D ligand expression following different types of stress and pattern recognition receptor (PRR) involvement.

Type of stress	Tissue	PRR (if any)	NKG2D ligand modulation	Reference
Rotavirus	Gut	TLR3	↑ RAE-1	(21)
*Lactobacillus*	Gut	–	↓ RAE-1	(22)
*Salmonella typhimurium*	Gut	TLR9	↓ RAE-1 ↓ MULT-1 ↓ H60	(24)
Ischemia	Kidney	TLR4	↑ RAE-1 ↑ MULT-1	(25)
Crohn’s disease (chronic inflammation)	Gut	–	↑ MICA	(31)
*Escherichia coli* (pathogenic)	Gut	–	↑ MICA	(32)
Celiac disease (chronic inflammation)	Gut	–	↑ MICA/B	(39)
ER stress	Gut	–	↑ MULT-1	(40)
*Akkermansia muciniphila*	Gut	–	↓ MICA/B	(56)
Oxidative	Lung	–	↑ MICA/B ↑ ULBP2	(59)
*Pseudomonas aeruginosa*	Lung	–	↑ RAE-1	(60)
Cigarette smoke	Lung	TLR3/7/9	↑ RAE-1 ↑ MICA	(62–65)

*↑, stands for upregulation; ↓, stands for downregulation on the indicated tissue; ER, endoplasmic reticulum*.

**Figure 1:**
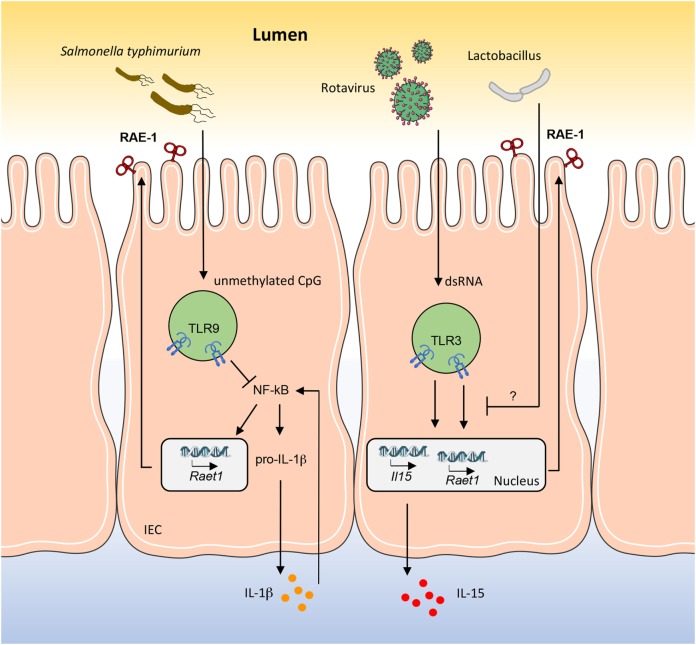
RAE-1 modulation in mouse intestinal epithelial cell (IEC) by gut pathogens and commensal bacteria. How RAE-1 (encoded by *Raet1*) is regulated after toll-like receptor (TLR) engagement is schematically depicted. The apical localization of RAE-1 is inferred based on its similarity to human ULBP1–3 structure. The mechanisms through which *Lactobacillus* strains are able to reduce the levels of RAE-1 and IL-15 are only partially known.

Rotaviruses are causative of intestinal alterations leading to diarrhea, and it has been reported that in mice viral dsRNA from rotavirus genome induces severe intestinal injury *via* TLR3 activation (20). Indeed, upon stimulation, TLR3 forces intestinal epithelial cells (IECs) to express both IL-15 and RAE-1, which promote mucosal damage by activating intraepithelial lymphocytes (IELs), in particular CD8αα^+^ T cells, engaging their NKG2D receptor (21). In this scenario, Tada and colleagues have shown that probiotics belonging to *Lactobacillus* strains are able to reduce the levels of IL-15 and RAE-1, and at the same time to increase the level of IL-10 in the intestine, thus performing immunomodulatory activities (22) (Figure 1). Interestingly, the NKG2D axis is modulated in an opposite way by TLR9 during *Salmonella typhimurium* infection. *S. typhimurium* is an important food-derived pathogen and unmethylated CpG-containing DNA from *S. typhimurium* is recognized by TLR9 (23). TLR9 triggering promotes the accumulation of IkBα, resulting in strong inhibition of the NF-kB pathway and thus controlling intestinal tissue inflammation. On the contrary, in TLR9-deficient mice, TLR9 signal deficiency releases its inhibition on NF-kB and leads to pro-IL-1β expression in IECs. In addition, lack of TLR9 signal causes activation of NLRP3 inflammasomes, resulting in pro-IL-1β processing and IL-1β secretion. Secreted IL-1β acts in an autocrine way stimulating IECs to expose NKG2D ligands (namely, RAE-1, MULT-1, and H60) and with a paracrine mechanism induces the expression of NKG2D on IELs (24). This positive loop augments the susceptibility of IECs to the cytotoxicity of IELs leading to the breaking of the epithelial barrier and to the spread of *S. typhimurium* infection (Figure 1).

TLR-dependent NKG2D ligand expression has also been observed in mouse kidney during renal ischemia–reperfusion injury (25). HMGB1 is a DNA-binding protein showing inflammatory function after ischemia–reperfusion injury by binding TLR4 (26–28). Chen and colleagues have reported that HMGB1 can induce RAE-1 and MULT-1 upregulation on injured kidney cells through a TLR4/MyD88-dependent signaling (25), suggesting a contribution of the NKG2D axis during tissue damage after ischemia. Accordingly, NK cell depletion has been demonstrated to ameliorate kidney damage following ischemia, with NK cells having a direct role in killing tubular epithelial cells *via* perforin secretion (29).

## Modulation of NKG2D Ligand Expression During Chronic Inflammation: The Gut Model

The gut system represents a peculiar challenge for the immune system, as it is continuously exposed to commensal bacteria and food antigens. IECs constitutively express MICA and MICB, even if at low levels (14, 30), while much less is known regarding ULBP proteins. In physiological conditions, this expression does not lead to an immune response, but during chronic inflammatory diseases, MICA/B-expressing cells become target of IELs, namely, intraepithelial NK cells, γδ T cells, and CD8^+^ αβ T cells. Although with different etiology, both Crohn’s disease and celiac disease are characterized by chronic inflammation. This type of stress strongly relies on the relationship between the environment and the immune system.

### Crohn’s Disease

MICA expression has been found significantly increased on IECs isolated from patients with Crohn’s disease, with higher levels in the macroscopically affected areas (31). Whether this upregulation is causative of the inflammation or a consequence of tissue damage is still unclear. Nevertheless, the upregulation of MICA has been associated with the expansion of a mucosal NKG2D^+^CD4^+^ T cell population able to promote a Th1 response, thus contributing to tissue inflammation and damage (31). There is evidence of a direct link between persistent MICA expression, innate lymphocyte activation and Th1 cytokine production in a model of pathogenic *Escherichia coli* infection (32), suggesting that this host–bacteria interaction may be relevant to the pathogenesis of Crohn’s disease, as adherent *E. coli* strains have been isolated from inflammatory bowel disease (IBD) patients (33, 34). The presence of adherent pathogenic *E. coli* triggers a rapid increase of MICA expression on the surface of intestinal cells after the interaction of the microbial adhesin AfaE with the cellular protein CD55, also known as the decay-accelerating factor (32). This event leads to NK cell activation with production of high levels of IFN-γ. As other human enteric pathogens, such as enteroviruses, use CD55 for cell entry (35, 36), it is possible to speculate that the *in loco* MICA overexpression occurring during chronic inflammation in unresolved infections contributes to lymphocyte activation associated with Crohn’s disease. Based on these findings, targeting the interaction between NKG2D and MICA may be seen as a promising strategy to reduce inflammation. Results of a randomized controlled trial for the use of an anti-NKG2D monoclonal antibody (NNC0142-0002) in active Crohn’s disease have reported no major improvement, but further investigations regarding dose ranging and dose regimen are needed (37). Furthermore, NKG2D ligands may have a wider role than stress sensors, contributing to the homeostatic control of the immune system, since a study by La Scaleia and colleagues has shown that NKG2D ligands, namely MICA/B and ULBP1–2, are upregulated not only on the epithelium of gut but also on the immune infiltrate in IBD lesions (38).

### Celiac Disease

Celiac disease is an immune-mediated disease characterized by damage to the small intestine in response to gluten exposure. Interestingly, there is evidence for a direct link between the expression of NKG2D ligands in the inflamed mucosa and cellular stress. Indeed, a similar MICA/B pattern of expression in the gut of celiac patients and in different *in vitro* models of cellular stress has been observed. In both cases, MICA/B were located in stress granules commonly observed after oxidative and endoplasmic reticulum (ER) stress (39). A recent study by Hosomi and colleagues has disclosed a molecular mechanism through which ER stress is associated with MULT-1 upregulation in IECs. In a mouse model of ER stress, the ER stress-related transcription factor C/EBP homology protein, a major component of the unfolded protein response, induces the transcription of the *Ulbp1* gene leading to MULT-1 cellular surface expression. MULT-1 upregulation has been linked to the activation of intraepithelial group 1 innate lymphoid cells (NK cells and ILC1) and innate-like T cells (such as CD8αα^+^ T cells), which contribute to mucosal inflammation in an NKG2D-mediated manner (40). Expression of NKG2D ligands in celiac disease can also be induced by cytokines, among which IL-15 seems to play a major role (41–45). Indeed, IL-15 has been shown to rapidly induce the expression of MICA and to relocate it from the cytosol to the surface membrane of enterocytes, where can be engaged by NKG2D-expressing IELs (3, 46, 47). On the other hand, MICA/B expression has been found also in the cytoplasm of intraepithelial and *lamina propria* lymphocytes (39). This intracellular localization of MICA/B in T cells during active celiac disease has been postulated to avoid overactivated T cell homeostatic regulation, thus contributing to tissue inflammation and damage.

An interesting issue to be considered for both Crohn’s disease and celiac disease regards NKG2D ligand polymorphism. MICA gene is highly polymorphic with more than one hundred alleles, which affect both RNA and protein expression levels (48). Some studies have assessed the prevalence of specific MICA alleles in IBD patients, with contrasting or not conclusive results (49). MICA allele*007 was associated with ulcerative colitis but not with Crohn’s disease in a British population (50), but this finding was not confirmed in a German cohort (51). MICA*008, instead, has been found overrepresented in celiac disease patients, but this could be ascribed to the linkage disequilibrium between HLA-B*08 and MICA*008 (52). Notably, MICA isoforms containing a methionine at position 129 bind NKG2D with high affinity, whereas those with a valine bind NKG2D with low affinity (53). Whether this diversity influences IBD is still unclear. A higher frequency of MICA-129met/met and a lower frequency of MICA-129val/met genotypes was observed in Spanish IBD patients compared with healthy controls (54). On the other hand, a study conducted in Chinese patients showed a higher frequency of the MICA-129val/val genotype in patients with ulcerative colitis (55).

The broad expression of NKG2D ligands on both epithelial cells and lymphocytes of the intestinal tract not only in pathological but also physiological conditions is intriguing but still unexplained. It may represent a state of alert ready to become active in response to stress, or, in more general terms, be part of tissue homeostasis regulation. On the other hand, it may be accountable for the state of permanent mild inflammation that characterizes the gut due to microbiota and food antigens. In support of the idea that the microbiota can be a main force driving the expression of NKG2D ligands on IECs is the observation that antibiotic administration and feeding are able to strongly modify NKG2D ligand expression (56). Germ-reducing conditions, such as ampicillin treatment, induce higher levels of NKG2D ligands, while food intake (i.e., xylooligosaccharides) or drug treatment supporting gut colonization by *Akkermansia muciniphila* decrease NKG2D ligand expression. An interesting association between the above conditions and the intestinal levels of IL-15 has been postulated, once again linking the inflammatory *milieu* to NKG2D ligands (56). Dietary contribution in the protection of gut inflammation has been investigated also in mice fed with a gluten-free diet. This food regimen leads to a reduced expression of NKG2D on DX5^+^ NK cells as well as to an increased number of CD8^+^ γδ T cells expressing TGF-β and the inhibitory receptor NKG2A, thus performing immunomodulatory functions (57, 58). Altogether, these findings reveal the importance of the NKG2D system in the homeostatic regulation of the intestinal mucosa.

## Role of NKG2D in the Airway Epithelium Following Stress

The respiratory tract is often under attack of environmental pathogens and is equipped with different populations of NKG2D-expressing lymphocytes. Bronchial airway epithelial cells constitutively express MICA, MICB, and ULBP1–4 transcripts, while cell surface expression is largely absent in normal conditions. However, upon oxidative stress, NKG2D ligands become visible at the protein level on the cellular membrane, with a molecular mechanism based on the ERK pathway (59). The capability of stressed airway epithelial cells to rapidly express NKG2D ligands suggests that they are able to directly engage and activate the immune system. This finding has been confirmed in mice with lung *Pseudomonas aeruginosa* infection. Acute infection stimulates the alveolar epithelium to express RAE-1, and epithelial cell death (likely due to the activation of cytotoxic NKG2D-bearing lymphocytes) is associated with increased bacterial clearance (60). Pulmonary *P. aeruginosa* infection is also associated with increased levels of IL-1β, TNF-α, and IFN-γ, which may be another effect of NKG2D engagement. Thus, NKG2D ligand expression leads to a competent host response that can be blocked with NKG2D-specific antibody (60).

The NKG2D system plays an important role in lung homeostasis also in the event of physicochemical stress, as demonstrated in chronic obstructive pulmonary disease (COPD). COPD is a severe form of airway epithelium inflammation largely due to cigarette smoke exposure. Cigarette smoke is known to induce necrotic and apoptotic cell death with concomitant nucleic acid release (61). This leads to TLR3/7/9 activation and, with an unknown mechanism, to RAE-1 expression on mucosal airway epithelial cells. The consequent inflammation and NKG2D-driven cytotoxicity is responsible for further tissue damage, worsening the initial cigarette smoke exposure. NK cells become hyperresponsive breaking the balance between injury and repair (62–64). Notably, NKG2D ligand overexpression in transgenic mice is sufficient to induce pulmonary emphysema, strongly suggesting that NKG2D/NKG2D ligand axis *via* cytotoxic lymphocyte activation plays a major role in alveolar epithelial injury (65). The strong relationship between NKG2D ligand expression and COPD has been further confirmed by the finding of enhanced MICA expression in the airway epithelium specimens from smokers (65).

## Concluding Remarks

Expression of NKG2D ligands can be achieved in several circumstances, ranging from viral and bacterial infections to physiochemical stress (Table 1). This versatility is needed to face a plethora of stressful stimuli with common pathways leading to the involvement of the immune system. As a result, intracellular insults are associated with intercellular responses, with great advantage for the whole organism. However, only recently the molecular mechanisms underlining these processes have been investigated, and many questions remain unanswered:

- Which ligand is displayed? Is this choice stress type dependent?
- Do different ligands engage the receptor in different ways?
- What is the contribution of NKG2D ligands to the pathogenesis of gut and lung inflammatory diseases? Does NKG2D ligand polymorphism play a role in IBD?

Indeed, both NKG2D and NKG2D ligand genes are highly polymorphic. This polymorphism has been associated with host–pathogen coevolution, with great relevance for viral infections, but functional consequences have also been described during hematopoietic transplantation and cancer immunosurveillance (48, 66, 67). The impact of this variability in the context of stress response and inflammation still needs to be fully elucidated.

Another important point to be considered is the strict polarity of epithelial tissues. In the gut, the apical side of the epithelial layer displays different functions from the basolateral side, where IELs are located. Human ULBP1–3 and murine RAE-1 are anchored to the cell membrane *via* a glycosylphosphatidylinositol molecule and thus are supposed to be trapped in the lipid raft-enriched apical side of epithelial cells (Figure 1), while MICA presents a basolateral-targeting motif in his structure (68). Hence, MICA expression is prone to a rapid recognition by NKG2D-bearing IELs, while ULBP1–3 and murine RAE-1 become available only in the event of epithelial polarity breaking, as result of infection or autoimmunity (69). Remarkably, MICA allele*008, due to its truncated cytoplasmic tail, lacks the basolateral-targeting motif and changes its localization, leading to hypothesize a different NKG2D engagement (68, 69). Furthermore, it is now clear that NKG2D ligand transcript and even protein expression does not always mean cell surface exposure, with obvious functional implications (70, 71).

Great interest is growing regarding the role of the microbiome in human pathophysiology, as commensal bacteria can establish an immune regulatory milieu in the intestine (72, 73). The articles here reported suggest that, beside the production of TGF-β and IL-10, modulation of the NKG2D axis is a key function in this context. Indeed, it is now emerging that the NKG2D receptor and NKG2D ligands not only function as stress sensor molecules but also play a pivotal role in shaping innate and adaptive lymphocyte populations, thus contributing to the homeostatic regulation of the mucosal immune system.

## Author Contributions

FA searched for literature articles; conceived and wrote the manuscript. ANS conceived and wrote the manuscript. ALS, CC, and GS revised and critically contributed to the manuscript drafting. FA, ALS, CC, GS, and ANS approved the final version of the manuscript.

## Conflict of Interest Statement

The authors declare that research was conducted in the absence of any commercial or financial relationship that could be construed as a potential conflict of interest.
